# Depletion of SNAP-23 and Syntaxin 4 alters lipid droplet homeostasis during *Chlamydia* infection

**DOI:** 10.15698/mic2020.02.707

**Published:** 2019-12-03

**Authors:** Tiago Monteiro-Brás, Jordan Wesolowski, Fabienne Paumet

**Affiliations:** 1 Department of Microbiology and Immunology, Thomas Jefferson University, Philadelphia, PA, USA 19107.; 2 Life and Health Sciences Research Institute (ICVS), School of Medicine, University of Minho, 4710-057 Braga, Portugal.; 3 ICVS/3B's, PT Government Associate Laboratory, 4710-057, Braga/Guimarães, Portugal.

**Keywords:** lipid droplets, Chlamydia, SNARE, intracellular bacteria, inclusion

## Abstract

*Chlamydia trachomatis* is an obligate intracellular pathogen that replicates inside a parasitic vacuole called the inclusion. The nascent inclusion is derived from the host plasma membrane and serves as a platform from which *Chlamydia* controls interactions with the host microenvironment. To survive inside the host cell, *Chlamydia* scavenges for nutrients and lipids by recruiting and/or fusing with various cellular compartments. The mechanisms by which these events occur are poorly understood but require host proteins such as the SNARE proteins (SNAP (Soluble N-ethylmaleimide-sensitive factor attachment protein) Receptor). Here, we show that SNAP-23 and Syntaxin 4, two plasma membrane SNAREs, are recruited to the inclusion and play an important role in *Chlamydia* development. Knocking down SNAP-23 and Syntaxin 4 by CRISPR-Cas9 reduces the amount of infectious progeny. We then demonstrate that the loss of both of these SNARE proteins results in the dysregulation of *Chlamydia*-induced lipid droplets, indicating that both SNAP-23 and Syntaxin 4 play a critical role in lipid droplet homeostasis during *Chlamydia* infection. Ultimately, our data highlights the importance of lipid droplets and their regulation in *Chlamydia* development.

## INTRODUCTION

*Chlamydia trachomatis* is the most common agent of bacterial sexually-transmitted infections with an estimated worldwide incidence of ~130 million cases per year [1, 2]. *C. trachomatis* is also the leading cause of preventable infectious blindness, called trachoma [3–5]. The rise in *C. trachomatis* infections, despite the availability of antibiotics, is compounded by the asymptomatic nature of disease progression. Consequently, untreated infections result in long-term complications, including pelvic inflammatory disease, ectopic pregnancy, and infertility [6]. Antibiotic treatment can also induce persistence, prolonging interactions between *Chlamydia* and its host, thus increasing the risk of developing chronic diseases [7–9].

*C. trachomatis* is an obligate intracellular pathogen with a unique developmental cycle consisting of distinct extracellular and intracellular forms [10, 11]. Elementary bodies (EBs) are the extracellular form and exhibit low metabolic activity, while reticulate bodies (RBs) are the metabolically active, replicative, but non-infectious, intracellular form. EBs promote their uptake into host epithelial cells by inducing local actin polymerization at the plasma membrane [12]. Once internalized, *C. trachomatis* remains inside a membrane-bound vacuole, called the inclusion. The nascent inclusion is derived from the plasma membrane but it acquires additional intracellular sources of lipids to support its considerable growth and expansion during *Chlamydia*'s life cycle. Ultimately, mature inclusions exceed the size of the host cell nucleus and occupy most of the cell's cytoplasm.

The intracellular survival of *Chlamydia* depends on its ability to control an array of interactions between the host and the inclusion, including contact with cellular organelles, which allow *Chlamydia* to scavenge for nutrients and lipids. Previous studies have demonstrated that *Chlamydia* acquires sphingomyelin and cholesterol by hijacking Golgi-derived vesicles that are destined for the plasma membrane [13–17]. In addition to the Golgi, the inclusion interacts with the endoplasmic reticulum (ER) [18–20], peroxisomes [21], and multivesicular bodies [22, 23]. *C. trachomatis* also utilizes host fatty acids (FA) to promote its growth. In eukaryotic cells, lipid droplets (LDs) are the primary site of FA storage and they have been shown to be involved in the intracellular development of *Chlamydia* [24–26]. *C. trachomatis* increases the LD content of its host cell during infection, and LD-like structures have also been reported in the lumen of chlamydial inclusions [25], suggesting that LDs are an important facet of *Chlamydia* infection.

*C. trachomatis* acquires resources from the host using multiple strategies, including diffusion mechanisms through transmembrane transporters, direct transfer of lipids at contact sites, and vesicle fusion [16, 27–31], the latter being mediated by SNARE proteins. The assembly of a specific vesicular SNARE (v-SNARE) with its cognate target SNARE (t-SNARE) complex into a stable four-helix bundle provides the energy necessary to disrupt and merge lipid bilayers during membrane fusion [32–35]. *Chlamydia* has been shown to control lipid acquisition by co-opting certain SNARE-mediated pathways. For instance, the siRNA-mediated depletion of Syntaxin 10 results in the retention of sphingomyelin in the inclusion while the depletion of VAMP-4 inhibits sphingomyelin trafficking to the inclusion [36–38]. In turn, by co-opting these pathways, *Chlamydia* enhances its survival inside the host cell [15, 16]. While these studies have begun to shed light on the role that SNARE proteins play during infection, the extent to which *Chlamydia* hijacks SNARE-mediated membrane fusion is unknown.

During internalization, the nascent inclusion membrane is formed from the host cell plasma membrane. Thus, this early membrane composition likely gives the inclusion distinct functional properties that would dictate interactions between the inclusion and the host cell. A number of SNARE proteins, including SNAP-23, Syntaxin 3, and Syntaxin 4, are located on the plasma membrane [39, 40]. Whether any of these SNAREs are retained on or excluded from the inclusion membrane is unknown.

In this study, we show that the SNAREs SNAP-23 and Syntaxin 4 are recruited to the chlamydial inclusion and that their depletion correlates with a decrease in infectious progeny, indicating that these plasma membrane SNAREs are important for *C. trachomatis* development. Interestingly, *C. trachomatis* infection does not affect constitutive secretion, which suggests that the function of both of these SNAREs is independent of their role in mediating membrane fusion at the plasma membrane. Instead, the loss of SNAP-23 and Syntaxin 4 results in a significant increase in *Chlamydia*-induced LDs, demonstrating that these SNAREs play an important role in controlling LD homeostasis during infection, which ultimately impacts *Chlamydia* development.

## RESULTS

### SNAP-23, Syntaxin 3, and Syntaxin 4 are recruited to the chlamydial inclusion

**Figure 1:**
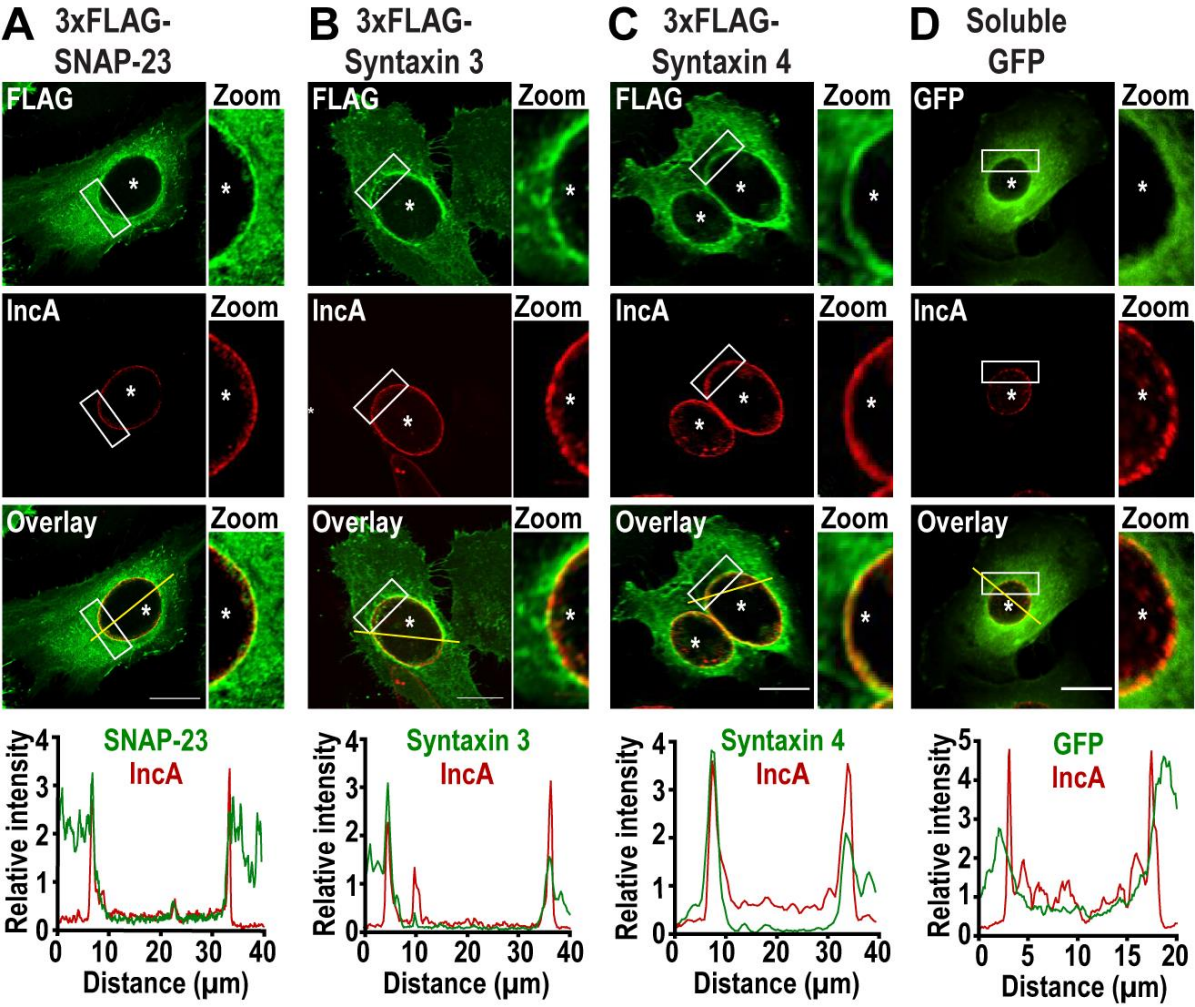
FIGURE 1: SNAP-23, Syntaxin 3, and Syntaxin 4 are recruited to the chlamydial inclusion. HeLa cells were infected with *C. trachomatis* at a MOI of 2 prior to being transfected with cDNA encoding **(A)** 3xFLAG-SNAP-23, **(B)** 3xFLAG-Syntaxin 3, **(C)** 3xFLAG-Syntaxin 4 or **(D)** GFP. At 24 h pi, the cells were fixed and labeled with anti-FLAG antibody (green) to label each SNARE and anti-IncA antibody (red) to identify the inclusion membrane. GFP-transfected cells (green) were only labeled with anti-IncA antibody (red). The boxed area of the inclusion membrane within each image is magnified to the right of each image (zoom). Asterisks denote inclusions. Scale bar = 20 μm. Line intensity scans (graphs) were conducted using ImageJ to determine if the FLAG or GFP signal was co-incident with IncA. Yellow line in the overlay image indicates the region of the line scan. Concomitant green (3xFLAG-SNARE or GFP) and red (IncA) peaks signify localization on the inclusion membrane.

To determine whether the plasma membrane SNAREs SNAP-23, Syntaxin 3, and Syntaxin 4 play a role during *Chlamydia* infection, we first assessed their localization during infection. To do so, we infected HeLa cells with *C. trachomatis* 4 h prior to transfection with cDNA encoding either 3xFLAG-SNAP-23, 3xFLAG-Syntaxin 3 or 3x-FLAG-Syntaxin 4. As a negative control, we transfected cells with a plasmid encoding soluble GFP. The cells were then fixed 24 h post infection (pi) and co-labeled with anti-FLAG antibody to determine the localization of each SNARE, as well as anti-IncA antibody to label the inclusion membrane. As shown in **Figure 1**, SNAP-23, Syntaxin 3, and Syntaxin 4, but not GFP, relocate to the inclusion where they are coincident with the inclusion membrane protein IncA **(Fig. 1**, zoom and line scans). The recruitment of these SNAREs was prevalent in cells expressing different levels of 3xFLAG-SNAREs (Fig. S1). Although previous studies have not detected endogenous or epitope-tagged Syntaxin 4 at the inclusion [38, 41], this may be due to method and timing of fixation. In our study, cells were fixed at 24 h pi with paraformaldehyde compared to 18 h pi with methanol in [38, 41]. We have found that the method and timing of fixation greatly influences the detection of SNARE proteins during *Chlamydia* infection (data not shown).

SNAP-23 encodes two light chains that interact either with the heavy chain Syntaxin 3 or Syntaxin 4 to form two distinct t-SNARE complexes [42]. Since these SNAREs are recruited to the inclusion, we tested whether *Chlamydia* infection impacts Syntaxin 3/SNAP-23 and Syntaxin 4/SNAP-23 t-SNARE complex formation. To do so, cells were infected with wild type (WT) *C. trachomatis* or mock infected and transfected at 4 h pi with a plasmid encoding 3xFLAG-SNAP-23. At 24 h pi, the samples were subjected to co-immunoprecipitation with anti-FLAG antibody and complex formation was analyzed by Western blot. As shown in Figure S2, comparable amounts of Syntaxin 3 and Syntaxin 4 co-immunoprecipitated with SNAP-23 in non-infected and *C. trachomatis*-infected cells.

Together, these results demonstrate that *C. trachomatis* recruits SNAP-23, Syntaxin 3, and Syntaxin 4 to the inclusion membrane without globally affecting interactions between SNAP-23 and its heavy chains.

### SNAP-23 and Syntaxin 4 are important for *Chlamydia* development

**Figure 2:**
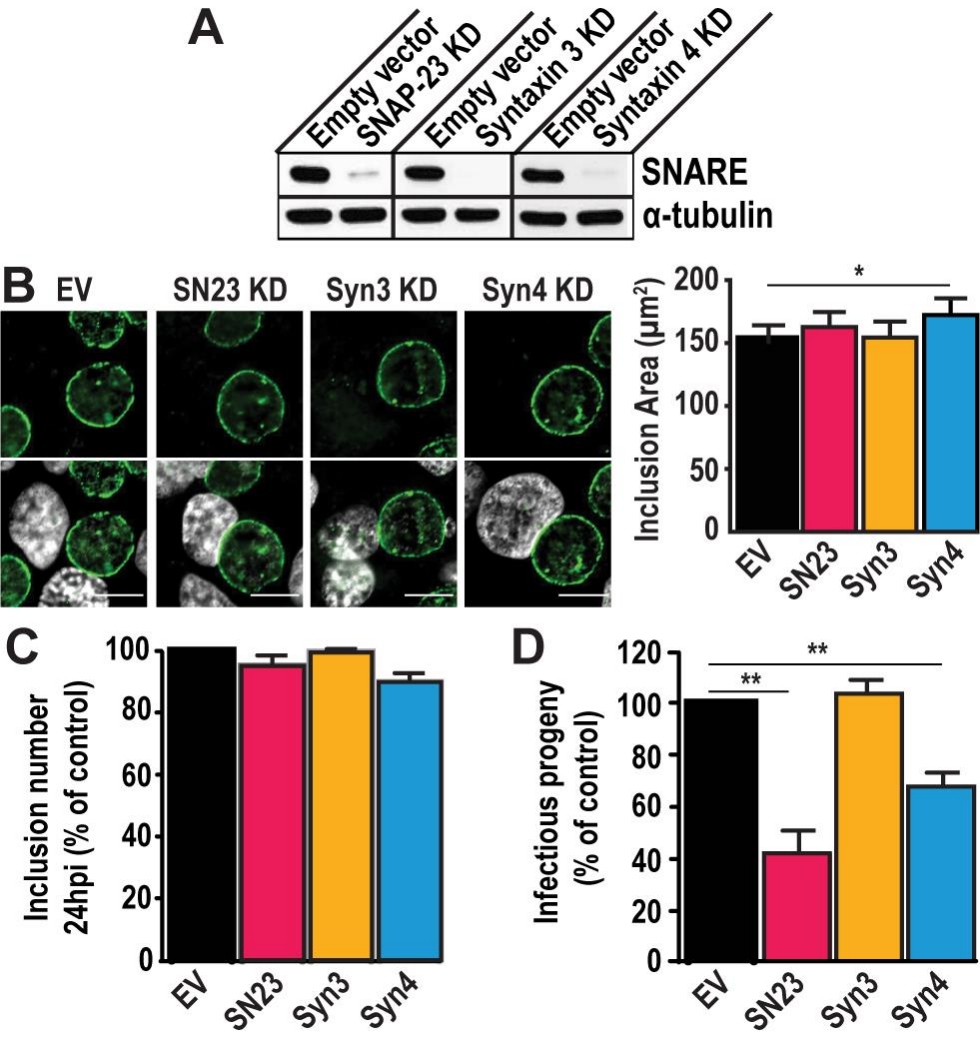
FIGURE 2: SNAP-23 and Syntaxin 4 regulate *Chlamydia* development. **(A)** Western blot analysis of protein levels in the indicated CRISPR knock down (KD) cell populations. SNARE corresponds to the t-SNARE in the KD cell line. Alpha-tubulin served as a loading control. **(B)** *Left*- The KD cell lines were infected with *C. trachomatis* L2 at a MOI of 0.5 for 24 h, then fixed and stained with anti-IncA antibody (green). Hoechst was used to label DNA (gray). EV = empty vector control, SN23 = SNAP-23, Syn3 = Syntaxin 3, Syn4 = Syntaxin 4, KD = knock down. Scale bar = 10 μm. *Right*- Graph displays the average inclusion area from three independent experiments ± the standard deviation. **(C and D)** Cells were infected with *C. trachomatis* at a MOI of 0.5. **(C)** At 24 h pi, cells were fixed and stained with anti-MOMP antibody. The number of inclusions per well was calculated to determine internalization and early inclusion development, assuming that one inclusion corresponded to an infection inititated by a single EB. Graph denotes the average of at least three independent experiments ± the standard deviation. For the purpose of comparison, the values obtained for the EV control were defined arbitrarily as 100% and represent 3.56x10^5^ ± 2.43x10^4^ inclusion forming units (IFU)/ml. Values for the KD cells were then normalized to the EV control. **(D)** At 46 h pi, cells were lysed and serially diluted on a fresh monolayer of EV control cells. Twenty-four hours later, the cells were fixed and stained with anti-MOMP antibody. Only dilutions reflecting a MOI < 1 were counted. Graph denotes the average fold change in IFU at T = 46 h compared to T = 0 h of at least three independent experiments ± the standard deviation. The fold change represents how many more IFU were present at T = 46 h compared to T = 0 h. For the purpose of comparison, the values obtained for EV control were defined arbitrarily as 100% and represent a fold change of 1,471.86 ± 153.4. Values for the KD cells were then normalized to the EV control. Asterisks denote significance, where (*) denotes a *p* value < 0.05 and (**) denotes a *p* value <0.01.

To test the importance of SNAP-23, Syntaxin 3, and Syntaxin 4 during infection, we depleted HeLa cells of either of these SNAREs using the CRISPR/Cas9 system (Fig. S3). These proteins were successfully knocked-down (KD) by at least 90 % compared to the empty vector control **(Fig. 2A)**. Note that all KD cells displayed the same growth kinetics as the empty vector control cell line, indicating that these SNARE proteins are not required for cell growth and survival (Fig. S4).

Using these KD cells, we tested the impact of SNAP-23, Syntaxin 3, and Syntaxin 4 depletion on inclusion development. To do so, we infected the KD cell lines with *C. trachomatis* for 24 h or 46 h. The cells were fixed and labeled with anti-IncA antibody to delineate the inclusion membrane, and the cells were visualized using immunofluorescence microscopy. As shown in **Figure 2B**, the inclusions that developed in the KD cell lines largely appear similar in size and shape compared to the empty vector control at 24 h pi. However, the inclusions that developed in Syntaxin 4 KD cells were 14% larger than those in empty vector control cells (172.2 μm^2^ versus 151.1 μm^2^). While this difference is statistically significant, it remains modest. We did not detect differences in inclusion area in SNAP-23 or Syntaxin 3 KD cells compared to the empty vector control cell line where the average inclusion size was 162.8 μm^2^, 154.6 μm^2^, and 151.1 μm^2^, respectively **(Fig. 2B**, graph). Similar results were observed at 46 h pi (Fig. S5). Together, these data suggest that SNAP-23, Syntaxin 3, and Syntaxin 4 do not play a major role in inclusion development.

Next, we determined whether *Chlamydia* grown in SNAP-23, Syntaxin 3 or Syntaxin 4 KD cells were less fit than those grown in the control cell line by quantifying the amount of infectious progeny generated from each of the KD cell lines. Knocking down SNAP-23, Syntaxin 3 and Syntaxin 4 did not impact internalization and early inclusion development as evidence by the number of inclusions detected at 24 h pi **(Fig. 2C)**. However, there was a ~60% decrease in infectious progeny generated from the SNAP-23 KD cell line and a ~35% decrease in the Syntaxin 4 KD cell line **(Fig. 2D)**. These data demonstrate that while SNAP-23 and Syntaxin 4 are not required for the establishment of infection, they do play a role in the later stage of progeny development.

### SNAP-23 and Syntaxin 4 play a non-canonical role during *Chlamydia* infection

The exocytic pathway is central to *Chlamydia* survival as the hijacking of Golgi-derived secretory vesicles provides much needed nutrients and resources for *Chlamydia* growth [14, 16]. To identify the specific role of SNAP-23 and Syntaxin 4 during *Chlamydia* infection, we assessed whether *Chlamydia* targets the plasma membrane SNAREs to hijack secretory cargo destined for the plasma membrane.

To do so, we measured whether *Chlamydia* infection interfered with constitutive secretion in the host cell using a HeLa cell line (HeLa C1) that stably expresses EGFP-tagged human growth hormone (hGH) containing tandem FKBP domains [43]. Under resting conditions, EGFP-hGH remained aggregated in the ER. Upon addition of D/D solubilizer, a disaggregation reagent, EGFP-hGH was released from the ER and ultimately secreted into the extracellular medium. We tracked the constitutive secretion of EGFP-hGH by measuring the EGFP signal that remained in the cell after the addition of the reagent to avoid dilution of the signal in the cell culture medium.

To test whether *Chlamydia* perturbs the constitutive secretory pathway, we infected HeLa C1 cells with *C. trachomatis.* At 22 h pi, we added the D/D solubilizer to release hGH from the ER and measured the secretion of EGFP-hGH for 2 h. We observed that the infected cells displayed similar kinetics of hGH secretion as the non-infected cells (Fig. S6A) and that EGFP-hGH was not detected inside of the inclusion in *Chlamydia*-infected cells (data not shown), indicating that hGH trafficking remains unperturbed during infection. These data are consistent with other model proteins used to track the secretory pathway during infection [17]. In light of these observations, the role of SNAP-23 and Syntaxin 4 during *Chlamydia* infection is likely independent of constitutive secretion. Note that after 22 h of infection, and before the addition of the D/D destabilizer, the EGFP signal was similar for infected and not infected cells (Fig. S6A, inset), indicating that the infection itself did not induce EGFP-hGH secretion or alter EGFP fluorescence.

*Chlamydia* also hijacks sphingomyelin (SM) and cholesterol from vesicles destined for the plasma membrane [13–17, 44]. Given the importance of the exocytic SNAREs in the fusion of secretory vesicles with the plasma membrane, we tested whether *Chlamydia* targets SNAP-23 and Syntaxin 4 to hijack sphingomyelin en route to the plasma membrane. To do so, we infected the KD cell lines with *C. trachomatis* for 20 h. A fluorescent analog of ceramide, NBD-C6-ceramide, was added to the cell culture medium, before being chased for 6 h. Ceramide is a SM precursor that is converted to SM in the Golgi and subsequently accumulates in the inclusion. Once the ceramide is processed by the host cell, the SM derived from it will retain the NBD signal and the excess ceramide is chased from the cell. In these experiments, SM localization was monitored by immunofluorescence microscopy and quantified by flow cytometry (Fig. S6B). We found that SM localized to the inclusion in all of the KD cell lines, indicating that there is no defect in SM trafficking to the inclusion in the absence of the plasma membrane SNAREs (Fig. S6B, images). Furthermore, the intensity of NBD fluorescence was similar in all of the KD cell lines compared to the empty vector control cells (Fig. S6B, graph), indicating that the amount of SM redirected to the inclusion is similar regardless of the absence of the exocytic SNAREs.

Altogether, these data suggest that the role of SNAP-23 and Syntaxin 4 during *Chlamydia* infection is independent of their canonical roles in mediating membrane fusion at the plasma membrane.

### SNAP-23 and Syntaxin 4 play a critical role in LD homeostasis during *Chlamydia* infection

In addition to mediating fusion events at the plasma membrane, SNAP-23 relocates from the plasma membrane and associates with cytoplasmic LDs where it participates in the homotypic fusion of LDs [45, 46]. Interestingly, *Chlamydia* infection increases the formation and production of LDs and LD-like structures have also been detected within the inclusion [25, 47]. Thus, we investigated whether SNAP-23, Syntaxin 3 or Syntaxin 4 play a role in LD dynamics during *Chlamydia* infection.

First, we determined whether the depletion of SNAP-23, Syntaxin 3 and Syntaxin 4 altered basal LD homeostatis in non-infected cells. Since HeLa cells do not store large quantities of LDs like adipocytes, we treated each KD cell line with oleic acid (OA) for 24 h. OA is a monounsaturated FA that is transported into the cell where it induces LD synthesis. The cells were then incubated with the fluorescent neutral lipid dye BODIPY 493/503 and their LD content was measured by flow cytometry (Fig. S7A). As expected, in all of the cell lines, the LD content of OA-treated cells was higher than untreated cells (Fig. S7B). Although the difference for the SNAP-23 KD cell line is statistically significant, it only corresponds to a modest ~12% increase (Fig. S7C). As for Syntaxin 3 and Syntaxin 4, we observed a minor decrease in LD induction of ~15% and 7%, respectively. In total, these data indicate that knocking down SNAP-23, Syntaxin 3 and Syntaxin 4 has a limited effect on LD synthesis and that OA induced LDs to a similar extent in the SNAP-23, Syntaxin 3 and Syntaxin 4 KD cell lines compared to the empty vector control cell line.

**Figure 3:**
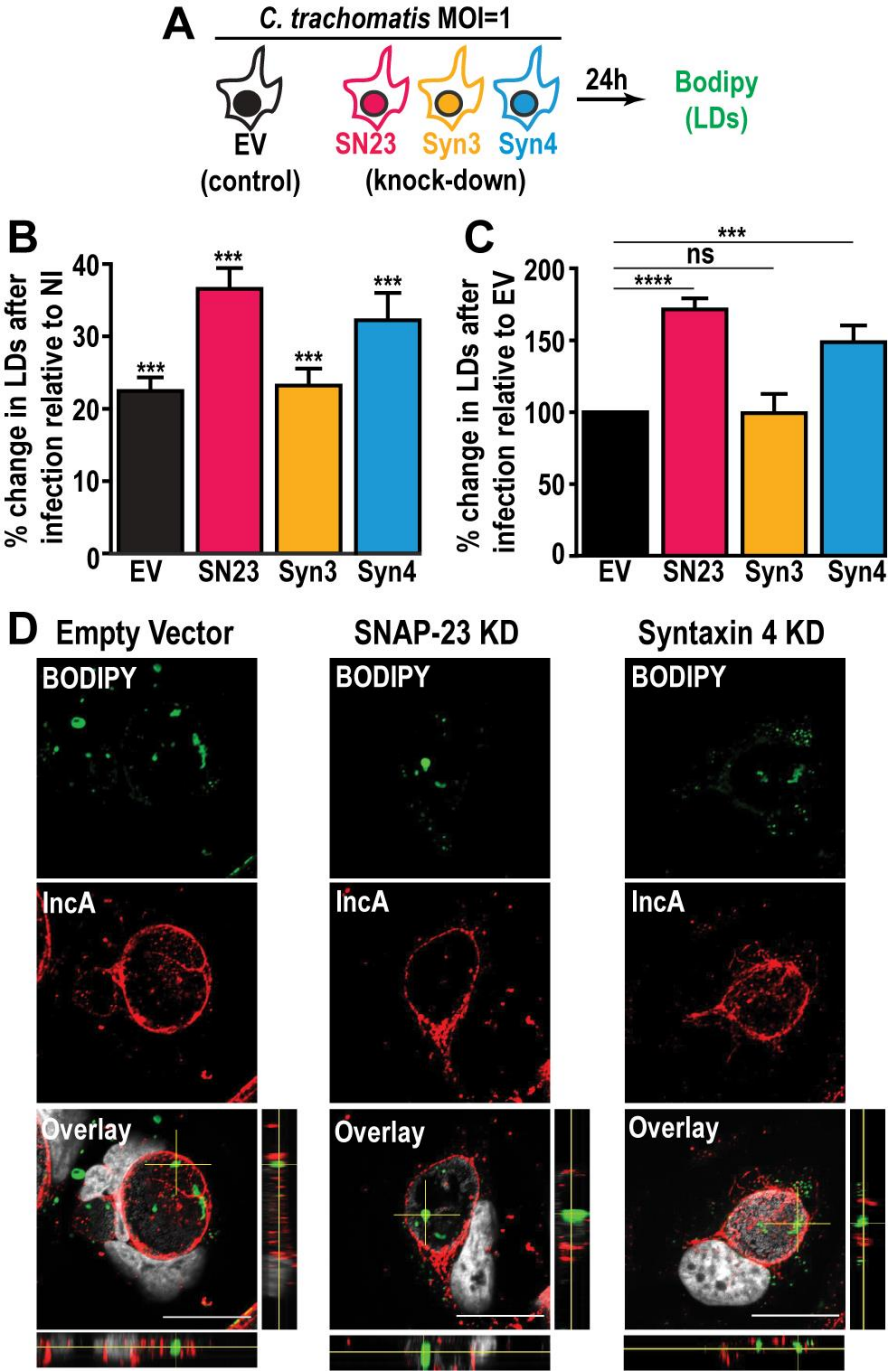
FIGURE 3: Depletion of SNAP-23 and Syntaxin 4 increases LD content during *Chlamydia* infection without affecting LD internalization into the inclusion lumen. **(A)** Experimental design. SN23 = SNAP-23, Syn3 = Syntaxin 3, Syn4 = Syntaxin 4, LDs = lipid droplets, MOI = multiplicity of infection. **(B and C)** Cells were either infected with mCherry-expressing *C. trachomatis* L2 at a MOI of 1 or mock-infected with DMEM for 24 h. The cells were then stained with BODIPY and fixed. BODIPY intensity was measured by flow cytometry. For the infected samples only the mCherry-positive population (infected cells) was measured. **(B)** Graph represents the average percent increase in BODIPY staining relative to non-infected cells from at least three independent experiments ± the standard deviation. Asterisk (***) denotes a *p* value < 0.001. **(C)** Graph represents the average increase in BODIPY staining relative to infected empty vector control cells from at least three independent experiments ± the standard deviation. The percent increase in BODIPY staining following infection for the empty vector control cell line was arbitrarily set at 100% and represents 121.41% ± 4.92. Values for the KD cells were then normalized to the EV control. Asterisk (***) denotes a *p* value < 0.001 and (****) denotes a *p* value <0.0001. ns = not significant. **(D)** The indicated cell lines were infected with *C. trachomatis* L2 at a MOI of 0.5 for 30 h. The cells were then fixed and stained with anti-IncA antibody (red) and BODIPY 493/503 (green). Hoechst was used to label DNA (gray). Images are taken from a single plane of confocal z-stacks. Orthogonal XZ and YZ views are shown next to and below the overlay image, respectively. Scale bar = 20 μm. KD = knock down.

Next, we investigated the impact of SNAP-23, Syntaxin 3, and Syntaxin 4 depletion on LD production during infection. To do so, we infected the empty vector control cells, as well as each KD cell line, with a strain of *C. trachomatis* that constitutively expresses mCherry, which allowed us to distinguish between infected and non-infected cells. At 24 h pi, the cells were incubated with BODIPY and analyzed by flow cytometry to measure their LD content **(Fig. 3A)**. In each of the cell lines, the infected cells displayed higher levels of BODIPY staining than the non-infected populations, confirming that *Chlamydia* infection triggers an increase in neutral lipid content **(Fig. 3B** and [47]). Next, we compared the degree to which *Chlamydia* increases neutral lipid content in each of the KD cell lines. Compared to the empty vector control cell line, we observed a ~71% and ~49% higher induction of LD content following infection in SNAP-23 and Syntaxin 4 KD cells, respectively **(Fig. 3C)**. In contrast, the induction of LDs by *Chlamydia* in Syntaxin 3 KD cells was ~1% lower than the empty vector control. These data indicate that SNAP-23 and Syntaxin 4, but not Syntaxin 3, are involved in LD homeostasis during infection. Furthermore, since the induction of LDs by OA-treatment in non-infected cells (Fig. S7C) was not affected by the absence of SNAP- 23 or Syntaxin 4, it is unlikely that the increase in LD content during infection **(Fig. 3C)** is due to a global dysregulation in LD synthesis. Rather, the role of SNAP-23 and Syntaxin 4 in the regulation of LDs is specific to *Chlamydia* infection.

During infection, *Chlamydia* internalizes LD-like structures into the inclusion lumen [25]. Thus, we determined whether the increase in LD content in SNAP-23 and Syntaxin 4 KD cells was due to an inability to internalize LDs into the inclusion. To accomplish this, we visualized BODIPY-stained structures inside of IncA-labeled inclusions generated in SNAP-23 and Syntaxin 4 KD cell line, compared to empty vector control cells. Using confocal microscopy **(Fig. 3D)** and 3-dimensional reconstruction (Fig. S8), we detected LD-like structures within inclusions generated in both of the KD cell lines, indicating that internalization of LDs into the lumen of the inclusion was intact and that these SNAREs are not essential for this process.

**Figure 4:**
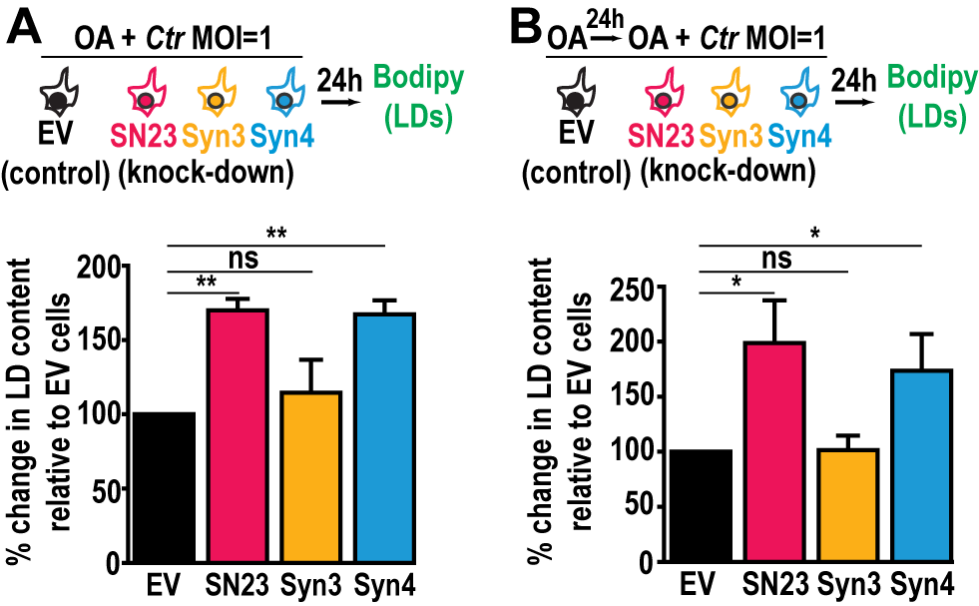
FIGURE 4. The addition of OA enhances the induction of LD content in SNAP-23 or Syntaxin 4 knock down cells during *Chlamydia* infection. Cells were **(A)** infected in the presence of OA for 24 h, or **(B)** pre-treated with OA for 24 h before infection. The cells were then stained with BODIPY, fixed, and the BODIPY intensity was measured by flow cytometry. For the infected populations, only the mCherry-positive cells were measured. Graphs represent the average increase in BODIPY staining following infection relative to empty vector control cells from at least three independent experiments ± the standard deviation. The percent increase in BODIPY staining following infection for the empty vector control cell line was arbitrarily set at 100% and represents **(A)** 174.72% ± 17.61% and **(B)** 181.63% ± 30.55%. Values for the KD cells were then normalized to the EV control. Asterisk (*) denotes a *p* value < 0.05 and (**) denotes a *p* value <0.01. ns = not significant. EV = empty vector control, SN23 = SNAP-23, Syn3 = Syntaxin 3, Syn4 = Syntaxin 4, LDs = lipid droplets. MOI = multiplicity of infection. *Ctr* = *Chlamydia trachomatis*.

The availability of FAs, which are incorporated into LDs, affects *Chlamydia* growth and development [48]. Since LD homeostasis is dysregulated during infection in SNAP-23 and Syntaxin 4 KD cells, we assessed the impact of increasing host cell FAs on *Chlamydia-*induced LDs in the SNAP-23 and Syntaxin 4 KD cell lines. This was tested in two configurations: (1) OA was added at the time of infection to assess the impact of supplemental LD synthesis on *Chlamydia*-induced LDs **(Fig. 4A**, schematic) and (2) OA was added 24 h prior to infection as well as at the time of infection to test the impact of increasing LD levels prior to infection **(Fig. 4B**, schematic). The addition of OA at the time of infection resulted in a substantial ~70% and ~67% increase in LD content compared to the empty vector control cell line in SNAP-23 and Syntaxin 4 KD cells, respectively **(Fig. 4A)**. This effect was further enhanced when cells were pre-treated with OA 24 h prior to the infection. In these conditions, we observed a ~98% and ~74% increase in neutral lipid content above the empty vector control cell line in SNAP-23 and Syntaxin 4 KD cells, respectively, compared to ~2% in the Syntaxin 3 KD cell line **(Fig. 4B)**. Together, these results demonstrate that both, SNAP-23 and Syntaxin 4, control LD homeostasis specifically during *Chlamydia* infection and that this effect can be boosted with exogenous FAs.

The dysregulation of LD homeostasis caused by knocking down SNAP-23 and Syntaxin 4 revealed an important pathway during infection as it correlates with a defect in *Chlamydia* development **(Fig. 2D)**. A potential explanation for the decrease in infectious progeny in the absence of SNAP-23 and Syntaxin 4 is that the increase in LD content during infection may be toxic to *Chlamydia*. To assess this possibility, we used OA to artificially increase the LD content in HeLa cells prior to infection. We then quantified early inclusion development at 24 h pi and the generation of infectious progeny at 46 h pi. OA treatment did not interfere with internalization and early inclusion development at 24 h pi (Fig. S9A), nor did it impact the generation of infectious progeny (Fig. S9B). These data indicate that simply increasing the LD content is not sufficient to interfere with *Chlamydia* growth and highlights a specific SNARE-dependent LD pathway induced by *Chlamydia* infection.

## DISCUSSION

LDs are heterogeneous storage organelles at the center of cellular lipid homeostasis [49]. They have a unique structure, consisting of a neutral lipid core surrounded by a phospholipid monolayer and have distinct protein compositions that likely reflect diverse functions within the cell [50]. LDs are dynamic organelles, that expand and contract according to the metabolic requirements of the cell. LDs originate from the ER, where they acquire most of their constituent molecules [50, 51]. After budding from the ER, LDs can grow through direct transfer of triacylglycerol from the ER membrane, local lipid synthesis [52], or through homotypic fusion [53]. LD homotypic fusion requires host SNARE proteins including SNAP-23, Syntaxin 5, and VAMP-4 [45]. Although the involvement of Syntaxin 4 in the LD pathway has not been previously shown, here we demonstrate that the loss of SNAP-23 or Syntaxin 4 results in the significant enhancement of *Chlamydia*-induced LDs. This effect is specific to *Chlamydia* infection since the induction of LDs by OA results in comparable levels of LDs in the empty vector control, SNAP-23, and Syntaxin 4 KD cell lines.

One possibility is that the increase in LD content observed in SNAP-23 and Syntaxin 4 KD cells during infection is due to a decrease in LD consumption by *Chlamydia* in these cells. *C. trachomatis* has been shown to translocate neutral lipids into the inclusion lumen [25, 47]. As the infection progresses, the amount of LD-like structures inside of the inclusion lumen appears to remain constant, suggesting that *Chlamydia* consumes these LDs as soon as they are engulfed [25]. *C. trachomatis* may require SNAP-23 on the inclusion membrane in order to hijack them, possibly through the recruitment of its cognate t-SNARE Syntaxin 4. If so, the loss of these SNAREs would impact the recruitment of LDs into the inclusion and ultimately impair the lipid composition of *Chlamydia*. Although we cannot exclude this possibility, immunofluorescence microscopy shows that LD-like structures are present inside of inclusions formed in SNAP-23 and Syntaxin 4 KD cells **(Fig. 3D** and S8), suggesting that some level of engulfment remains and that this alone does not account for the significant growth defect observed in these cell lines.

An alternative possibility is that *C. trachomatis* infection stimulates a pathway in which SNAP- 23 and Syntaxin 4 control the formation and/or hijacking of a particular subset of LDs. *Chlamydia-*induced LDs likely have a distinct composition from OA-induced LDs. In fact, it has been shown that *Chlamydia* secretes LD-binding proteins called Lda [47]. While Lda1 and Lda3 bind mammalian LDs in the cytosol, Lda2 only binds LDs induced during infection, highlighting the distinct composition of LDs generated during *Chlamydia* infection. SNAP-23 and Syntaxin 4 could influence the formation of these types of *Chlamydia*-induced LDs. Ultimately, their depletion would lead to the accumulation of LDs that are unable to be metabolized by *Chlamydia*, which would then negatively impact *Chlamydia* fitness. This hypothesis is supported by several observations: i) SNAP-23 and Syntaxin 4 are specifically involved in the formation of *Chlamydia*-induced but not OA-induced LDs, supporting the existence of multiple LD synthesis pathways **(Fig. 3B**, **4**, and S7); ii) the decrease in infectious progeny generated in SNAP-23 and Syntaxin 4 KD cells demonstrate that *Chlamydia* replication is impaired **(Fig. 2D)**, even though LD-like structures are observed inside the inclusion lumen **(Fig. 3D** and S8); and iii) *Chlamydia* grown in cells in which LDs were induced by OA are not replication defective (Fig. S9), indicating that a surplus of OA-induced LDs is not toxic.

In summary, our data show that the loss of both SNAP-23 and Syntaxin 4 results in an aberrant increase in LD production during *Chlamydia* infection. *Chlamydia*, in turn, exhibits a decrease in infectious progeny, likely due to the altered composition of the LDs generated in these cells in the absence of these two SNAREs. Additional studies are necessary to compare the composition of *Chlamydia*-induced LDs versus OA-induced LDs. Ultimately, identifying the molecular components involved in this pathway will shed light on how both of these SNAREs regulate LD homeostasis during *Chlamydia* infection.

## MATERIALS AND METHODS

### Cell culture and *Chlamydia* strains

HeLa cells (ATCC CCL-2) were cultured in Dulbecco's modified Eagle's medium (DMEM) supplemented with 10% fetal bovine serum (FBS), 2 mM L-glutamine, 1 mM sodium pyruvate, MEM non-essential amino acids and 10 μg/mL gentamicin. HeLa C1 cells expressing human growth hormone fused to EGFP and tandem mutated FKBP domains were a gift from Dr. Andrew Peden (University of Sheffield) [43]. *C. trachomatis* serovar L2 strain 434/Bu and a recombinant strain constitutively expressing mCherry were kindly provided by Dr. T. Hackstadt (NIH, NIAID) [54].

### Antibodies and dyes

The following primary antibodies were used: rabbit anti-IncA (gift from T. Hackstadt), goat anti-MOMP (Virostat), rabbit anti-Syntaxin 3 (Synaptic Systems), rabbit anti-Syntaxin 4 (Sigma), rabbit anti-SNAP-23 (Sigma), mouse anti-alpha tubulin (Sigma), mouse anti-FLAG (Sigma, M2), chicken anti-HSP70 (StressMarq). Goat anti-rabbit IgG-Alexa Fluor 488, goat anti-rabbit IgG-Alexa Fluor 647, goat anti-mouse IgG-Alexa Fluor 488, goat anti-rabbit IgG-Alexa Fluor 555, donkey anti-goat IgG-Alexa Fluor 488, donkey anti-rabbit IgG-, anti-mouse IgG-, anti-goat IgG-, and anti-chicken IgY-horseradish peroxidase (HRP)-conjugated secondary antibodies, Hoechst, and NBD-C6-ceramide were purchased from Invitrogen. BODIPY 493/503 and CFSE Cell Proliferation Kit were purchased from ThermoFisher.

### Plasmid construction and genome editing

Polymerase chain reactions (PCR) were performed with High Fidelity polymerase (Accuris) according to manufacturer's protocols. FastDigest restriction enzymes, FastAP phosphatase, and T4 DNA ligase were purchased from ThermoFisher. The DH5α *E. coli* strain was used for plasmid amplification and isolation. All primers are listed in Table S1. Human SNAP-23 was amplified from pEGFP-human SNAP-23 (Addgene) using the oligonucleotides FO1294 and FO1296. Rat Syntaxin 3 was amplified from rat Syntaxin 3-EGFP (FD269) using the oligonucleotides FO1327 and FO1328. Rat Syntaxin 4 was amplified from rat Syntaxin 4-EGFP (FD270) using the oligonucleotides FO1329 and FO1330. Note that both rat Syntaxin 3 and Syntaxin 4 are 97.22% and 89.23% identical to human Syntaxin 3 and Syntaxin 4, respectively (Fig. S10) and are commonly used to replace human Syntaxins [55, 56]. PCR fragments were cloned into the EcoRI and XhoI sites of pCMV-Tag2B (gift from Paul Roche, NIH), which was modified to contain a 3xFLAG tag instead of a single FLAG tag. DNA sequences were confirmed by Sanger sequencing (GenScript).

Guide RNA and confirmation primers were designed using the exon-intron structures available at GenBank. These guide RNAs were then analysed for their efficiency and theoretical off-target effects, using the *CRISPOR* [57] and the *Broad Institute sgRNA designer* [58] databases with default cut-offs. Only guideRNAs with appropriate scores from these databases were selected. Guide RNAs were cloned into the pSpCas9(BB)-2A-GFP (PX458) vector (provided by S. Kim) and confirmed by Sanger sequencing. HeLa cells were seeded in 100 mm tissue culture dishes 24 h prior to being transfected with 6 μg of plasmid at a 1 μg:1 μl ratio using the Continuum Transfection Reagent (Gemini Bio-products) and incubated for 24 h at 37°C and 5% CO_2_. Cells transfected with the vector encoding only Cas9-GFP (no guide RNA) were used as a control. The cells were then sorted based on GFP expression, using flow cytometry (BD FACSAria II). To confirm successful targeting of the guide RNA and Cas9 cleavage, genomic DNA was extracted from each cell population and a region corresponding to the predicted Cas9 cut site was PCR amplified using the confirmation primers. For each reaction, 2 μg genomic DNA was used. The full PCR reaction was run on a 1% agarose gel and the amplicon band was cut and purified. Cas9 activity was assessed by digesting the amplicon with 10 U of T7E1 endonuclease at 37°C for 1 h (Fig. S3). Cell lines with productive Cas9 activity were analyzed by Western blot to determine the level of target protein knock down (see below). The protocol was repeated until protein levels were reduced by at least 90%. Each knock down cell line was maintained as a population to prevent any potential off-target or clonal effects.

### Western blotting

Samples were lysed in cold lysis buffer (50 mM Tris, 100 mM NaCl, 2 mM MgCl_2_, 1% Nonidet- P40, 10% glycerol, pH 7.5 supplemented with 2 mM phenylmethanesulfonyl fluoride, 2 μM pepstin A, 10 μM leupeptin, 10 mM NaF, and 5.4 mM Na_3_VO_4_) for 1 h on ice. Lysates were clarified by centrifugation and protein concentrations were measured using the Pierce 660 nm Protein Assay (ThermoFisher). The samples were diluted in loading buffer (50 mM Tris, 2% sodium dodecyl sulfate, 10% glycerol, 0.3 mM bromophenol blue, 2.5% β-mercaptoethanol), separated on 12% Tris-glycine SDS-PAGE gels, and transferred at 100 V to polyvinylidene difluoride membrane (Millipore) for 1 h at 4°C. The membranes were rinsed with wash buffer (25 mM Tris, 150 mM NaCl, pH 7.5, 0.1% Tween-20) and blocked with wash buffer containing 3% bovine serum albumin (BSA) and 0.05% NaN_3_ for 1 h at room temperature (RT). Membranes were probed with the indicated primary antibodies diluted in the blocking buffer overnight at 4°C. The membranes were then washed several times with wash buffer and incubated with HRP-conjugated secondary antibodies diluted in wash buffer containing 0.5% milk for 1 h at RT. Following several washes, the blots were revealed with SuperSignal West Dura Extended Duration Substrate (ThermoFisher) and autoradiography film.

### Immunofluorescence staining

Cells were fixed either with ice-cold methanol or 4% paraformaldehyde (PFA) for 20 min at RT and washed in IF buffer (0.9 mM CaCl_2_, 0.5 mM MgCl_2_, 150 mM NaCl, 100 mM glycine, 25 mM HEPES, pH 7.5). The cells were permeabilized and blocked in blocking buffer (0.1% Triton X-100, 10% normal goat serum in IF buffer) for 30 min at RT. Coverslips were then incubated with rabbit anti-IncA antibody (1:500) and/or mouse anti-FLAG (M2 Sigma, 1:100) diluted in blocking buffer for 1 h at RT. The coverslips were then washed several times with IF Buffer containing 0.1% Triton X-100 and incubated with goat anti-rabbit IgG Alexa Fluor 555 and goat anti-mouse IgG Alexa Fluor 488-conjugated secondary antibodies (Invitrogen, 1:500) and 1 μg/ml Hoechst (Invitrogen) diluted in blocking buffer for 1 h at RT. Coverslips were washed with IF buffer and mounted with Prolong Glass Antifade reagent (Invitrogen).

### SNARE recruitment to the inclusion

HeLa cells were seeded onto coverslips in a 24-well plate at a density of 3x10^4^ cells per well 24 h prior to infection. The cells were infected with *C. trachomatis* L2 at a multiplicity of infection (MOI) of 2 by centrifugation at 1,000 *xg* for 1 h at 4°C and then transitioned to 37°C and 5% CO_2_ for 3 h. The infected cells were then transfected with 25 ng of each plasmid supplemented with 225 ng of empty pcDNA3.1+ plasmid at a 1 μg of DNA:1 μl of Continuum transfection reagent (Gemini Bioproducts) ratio and incubated for an additional 21 h at 37°C and 5% CO_2_. At 24 h pi the cells were fixed and stained as described above. Images were acquired with a Nikon A1R+ confocal microscope with 60x oil immersion lens and Nikon NIS Elements software. Contrast enhancement and image cropping, as well as line scans were conducted using ImageJ (NIH).

### Inclusion size analysis

HeLa cells were seeded onto coverslips at a density of 1x10^5^ cells per well in a 24-well plate 24 h prior to infection. The cells were infected with *C. trachomatis* L2 at a MOI of 0.5 by centrifugation at 1,000 *xg* for 1 h at 4°C. The cells were then transitioned to 37°C and 5% CO_2_ for 24 h or 46 h. The cells were fixed and stained as described above. Images were acquired using a Nikon TiE inverted immunofluorescence microscope with a 60x oil immersion lens and Nikon NIS Elements AR 4.2 software. Contrast enhancement and image cropping were performed using ImageJ (NIH). Inclusion area was acquired by tracing the outline of the inclusion using NIS Elements software. A minimum of 100 inclusions per sample were measured for each experiment.

### Progeny analysis

HeLa cells were seeded at a density of 1x10^5^ cells per well in a 24-well plate 24 h prior to infection. Cells were infected with *C. trachomatis* L2 at a MOI of 0.5 by centrifugation at 1,000 *xg* for 1 h at 4°C. At 24 h pi, one set of cells were fixed and stained with anti-IncA antibody as described above to determine the inclusion forming units (IFU) at infection (T = 0 h). At 46 h pi, the other set of cells were lysed in sterile water. The lysates were serially diluted in HBSS and used to infect a new monolayer of empty vector control cells. The cells were then fixed 24 h pi and labeled with anti-IncA antibody as described above. Images were acquired and analyzed using a Nikon TiE inverted immunofluorescence microscope with 20x lens and Nikon NIS Elements AR 4.2 software. Image processing was conducted using ImageJ (NIH). Each condition was prepared in triplicate for each experiment, and a minimum of 20 images per well were counted. The number of inclusions in each well was then calculated considering the dilution factor and the surface area of the well. The fold change in IFU was calculated by dividing the number of IFU obtained from T = 46 h by the IFU at T = 0 h. The resulting ratio denotes how many more IFU were found at T = 46 h compared to T = 0 h. For comparison purposes, all values were then normalized to the empty vector values.

### Constitutive secretion assay

HeLa C1 cells were seeded at a density of 8.8x10^3^ cells per well in a 96-well plate 24 h prior to infecting with *C. trachomatis* L2 at a MOI of 2. The infection was synchronized by centrifugation at 1,000 *xg* for 1 h at 4°C and the cells were incubated at 37°C and 5 % CO_2_ for 22 h. EGFP human growth hormone (hGH) was released from the ER with 0.7 μM D/D solubilizer dissolved in ethanol (ClonTech) or ethanol alone (control). At the indicated time points, the wells were washed with HBSS to remove secreted EGFP-hGH. The EGFP-hGH remaining in the cells was measured on SpectraMax M2 spectrophotometer (Molecular Devices). EGFP was excited at 460 nm and emission was read at 510 nm. HeLa cells not expressing EGFP-hGH were used to control for autofluorescence, which was subtracted from all wells containing HeLa C1 cells. EGFP-hGH secretion was calculated using the following formula:

% EGFP-hGH secretion = 100% − [(A – A1) / (B − B1) × 100]

Where, A = EGFP intensity of HeLa C1 well containing D/D solubilizer, A1 = EGFP intensity of HeLa well containing D/D solubilizer, B = EGFP intensity of HeLa C1 well containing ethanol, B1= EGFP intensity of HeLa well containing ethanol.

### Ceramide acquisition

HeLa cells were seeded onto coverslips or onto wells at a density of 1x10^5^ cells per well in a 24-well plate 24 h prior to infection. Cells were infected with *C. trachomatis* L2 constitutively expressing mCherry at a MOI of 0.5 for flow cytometry or with WT *C. trachomatis* L2 at a MOI of 1 for immunofluorescence analysis. The infection was synchronized by centrifugation at 1,000 *xg* for 1 h at 4°C. The cells were transitioned to 37°C and 5% CO_2_ for 20 h. The cells were incubated with 5 μM NBD-C6-ceramide for 30 min at 37°C. The cells were washed with HBSS and incubated with 0.34% FA-free BSA in serum-free DMEM for 6 h to allow for back-exchange. For microscopy analysis, the coverslips were washed several times with HBSS, stained with 1 μg/ml of Hoechst (Invitrogen) for 5 min at 37°C and mounted for live cell imaging with HBSS. Images were acquired and processed using a Nikon TiE inverted immunofluorescence microscope with a 60x oil immersion lens and Nikon NIS Elements AR 4.2 software. Contrast enhancement and image cropping were conducted using ImageJ (NIH). For flow cytometry, the cells were trypsinized into suspension and fixed in 4% PFA in HBSS for 10 min at RT. Each sample was resuspended in FACS Buffer (25 mM HEPES, 1 mM EDTA, 1% FBS in phosphate buffered saline [PBS], pH 7.0) and analyzed on LSRFortessa flow cytometer (BD Biosciences).

### Lipid droplet quantification assay

HeLa cells were seeded at a density of 6x10^4^ cells per well in a 24-well plate. To pre-treat cells with OA, cells were incubated with 200 μM of OA diluted in PBS containing 10% FA-free BSA for 24 h. DMSO was used as the control. The cells were then infected with *C. trachomatis* L2 constitutively expressing mCherry at a MOI of 1 by centrifugation at 1,000 *xg* for 1 h at 4°C or mock-infected with DMEM. For OA treatment at the time of infection, 200 μM of OA or DMSO was added after centrifugation. The cells were then transitioned to 37°C and 5% CO_2_ for 24 h. The cells were stained with 50 μM BODIPY 493/503 (ThermoFisher) for 30 min at 37°C and washed several times with HBSS. The cells were trypsinized into a single cell suspension and fixed in 4% PFA in HBSS for 10 min at RT. Each sample was then resuspended in FACS Buffer and analyzed on a LSRFortessa flow cytometer (BD Biosciences). For each cell line, a ratio was obtained for the BODIPY intensity in the corresponding control cells with that of the treated cells (i.e. not infected:infected or DMSO:OA). This ratio for the EV control cells was considered 100% and all values were normalized to the EV ratio for each experiment. Each experiment was independently performed at least 3 times in triplicate.

### LD analysis inside inclusion

HeLa cells were seeded at a density of 4x10^4^ cells per coverslip in a 24-well plate 24 h prior to infection. The cells were infected with *C. trachomatis* L2 at a MOI of 0.5 by centrifugation at 1,000 *xg* for 1 h at 4°C. The cells were then transitioned to 37°C and 5% CO_2_. At 30 h pi, cells were fixed with 3% PFA and 0.025% of glutaraldehyde in PEMS buffer (80 mM PIPES, pH 7.0, 5 mM EGTA, 2 mM MgCl_2_, 50 mM sucrose) for 20 min at RT. The coverslips were washed with IF buffer containing 100 mM glycine. The cells were permeabilized with PBS containing 0.2 M glycine, 30 mg/mL BSA and 0.01% saponin for 45 min at RT and then blocked with blocking buffer (10% horse serum, 0.05% NaN_3_ in IF Buffer) for 30 min at RT. Coverslips were washed with IF buffer and then incubated with anti-IncA antibody (1:500) diluted in PBS containing 0.01% saponin and 1 mg/mL of BSA for 1h at RT. The coverslips were then washed several times with IF Buffer and incubated with goat anti-rabbit IgG Alexa Fluor 647-conjugated secondary antibody (Invitrogen, 1:500), 1 μg/mL Hoechst (Invitrogen) and 12.5 μM BODIPY 493/503 (ThermoFisher) in PBS containing 0.01% saponin and 1 mg/mL of BSA for 1 h at RT. Coverslips were mounted with Prolong Glass Antifade reagent (Invitrogen). Images were acquired with a Nikon A1R+ confocal microscope with 60x oil immersion lens and Nikon NIS Elements software. Contrast enhancement and image cropping, as well as 3D reconstruction were conducted using ImageJ (NIH).

### Cell Division Assay

HeLa cells were stained with 5 mM carboxyfluorescein succinimidyl ester (CFSE) (Cell Trace Cell Proliferation Kit, Invitrogen) and seeded in 6-well plates at 1x10^5^ cells per well and incubated at 37°C with 5% CO_2_ for 24 h, 48 h, 72 h and 96 h. At each timepoint, the cells were collected and resuspended in FACS Buffer. CFSE intensity was measured on a LSRFortessa flow cytometer (BD Biosciences).

### Immunoprecipitation

HeLa cells were seeded onto 150 mm tissue culture dishes at a density of 3.6x10^6^ cells per dish 24 h prior to infection. The cells were infected with *C. trachomatis* L2 at a MOI of 5 or mock infected with HBSS by absorption for 2 h at 37°C rocking and then transitioned to 37°C and 5% CO_2_ for 2 h. Both cell populations were then transfected with 2 μg of 3xFLAG-SNAP-23 DNA supplemented with 7 μg of empty pcDNA3.1+ plasmid at a 1 μg of DNA:1 μl of Continuum transfection reagent (Gemini Bio-products) ratio and incubated for an additional 20 h at 37°C and 5% CO_2_. At 24 h pi the cells were lysed in ice-cold lysis buffer (50 mM Tris, pH 8.0, 150 mM NaCl, 10 mM EDTA, 5% glycerol, 1% Triton X-100) supplemented with 250 U/ml of Turbonuclease (Accelagen), 10 mM NaF, 5.4 mM Na_3_VO_4_, and protease inhibitor cocktail (APExBIO) for 1 h on ice. Samples were clarified by centrifugation at 20,000 *xg* for 30 min at 4°C and then incubated with 50 μl of settled Protein G Plus agarose (Calbiochem) for 30 min at 4°C. Pre-cleared samples containing 6 mg of total protein were then incubated with 30 μg of mouse anti-FLAG antibody (Sigma, M2) overnight at 4°C rotating (8 rpm). Antibody-antigen complexes were captured with 20 μl of settled Protein G Plus agarose at 4°C for 2 h, washed with lysis buffer, and eluted with SDS-PAGE sample buffer. Eluates and lysates (40 μg) were analyzed by Western blot.

### Statistics

A two-tailed student t-test was used to determine statistical differences between the means of two populations from at least three independent experiments. Statistical significance was assumed at *p* values < 0.05.

## SUPPLEMENTAL MATERIAL

Click here for supplemental data file.

All supplemental data for this article are available online at www.microbialcell.com.

## Abbreviations:

EB: – elementary body,
ER: – endoplasmic reticulum,
FA: – fatty acid,
hGH: – human growth hormone,
KD: – knock down,
LD: – lipid droplet,
MOI: – multiplicity of infection,
OA: – oleic acid,
pi: – post infection,
RT: – room temperature,
SM: – sphingomyelin,
SNAP: – soluble N-ethylmaleimide-sensitive factor attachment protein,
SNARE: – SNAP receptor,
t-SNARE: – target SNARE,
v-SNARE: – vesicular SNARE,
WT: – wild type.
