# Supplementary material for: Depletion of SNAP-23 and Syntaxin 4 alters lipid droplet homeostasis during Chlamydia infection
Source: Microb Cell. 2019 Dec 3;7(2):46–58. doi: 10.15698/mic2020.02.707 (PMC6993123; doi:10.15698/mic2020.02.707)
Supplement: Supplementary file 1 [file mic-07-046-s01.pdf]

SUPPELEMENTAL MATERIAL

Depletion of SNAP-23 and Syntaxin 4 alters lipid droplet homeostasis during *Chlamydia* infection

Tiago Monteiro-Brás[1,2,3], Jordan Wesolowski[1,*] and Fabienne Paumet[1,*]

[1] Department of Microbiology and Immunology, Thomas Jefferson University, Philadelphia, PA, USA 19107.
[2] Life and Health Sciences Research Institute (ICVS), School of Medicine, University of Minho, 4710-057 Braga, Portugal.
[3] ICVS/3B's, PT Government Associate Laboratory, 4710-057, Braga/Guimarães, Portugal.
* Corresponding Authors:
Fabienne Paumet, 233 South 10th Street, Bluemle Life Sciences building room 750,
Philadelphia, PA 19104; Phone: 215-503-8567; Fax: 215-923-6852; E-mail: Fabienne.Paumet@Jefferson.edu;
Jordan Wesolowski, 233 South 10th Street, Bluemle Life Sciences building room 750,
Philadelphia, PA 19104; Phone: 215-503-8566; Fax: 215-923-6852; E-mail: Jordan.Wesolowski@Jefferson.edu

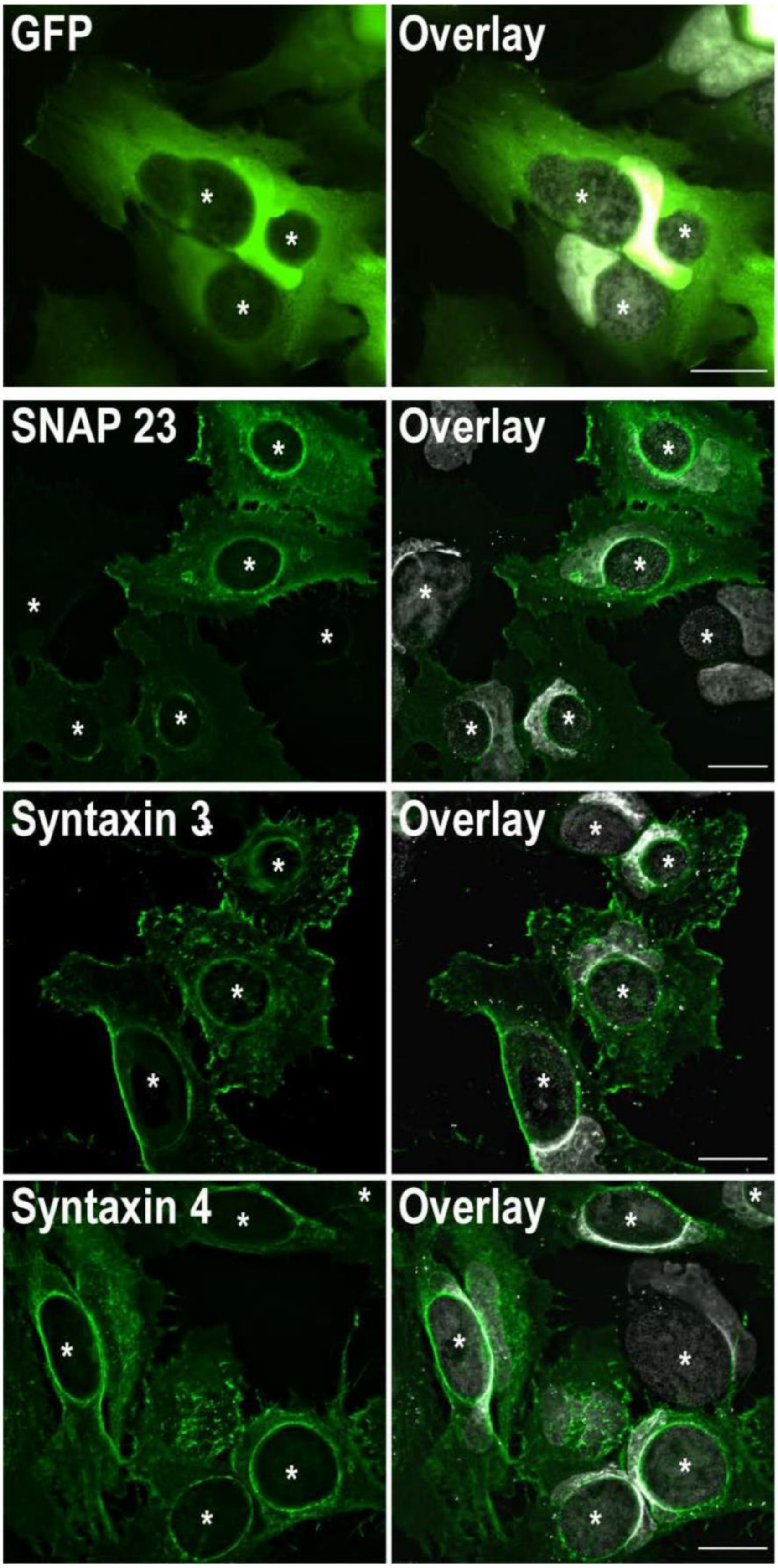

FIGURE S1: Recruitment of SNAP-23, Syntaxin 3, and Syntaxin 4 to the inclusion is prevalent in cells with different expression levels. HeLa cells were infected and transfected as in Figure 1. At 24 h pi the cells were fixed and labeled with anti-FLAG antibody (green) to label each SNARE and Hoechst (gray) to label DNA. GFP-transfected cells (green) were only labeled with Hoechst. Asterisks denote inclusions. Scale bar = 20 µm. Note that 3xFLAG-SNAP-23, 3xFLAG-Syntaxin 3, and 3xFLAG-Syntaxin 4, but not GFP, are enriched in a ring pattern around the inclusion.

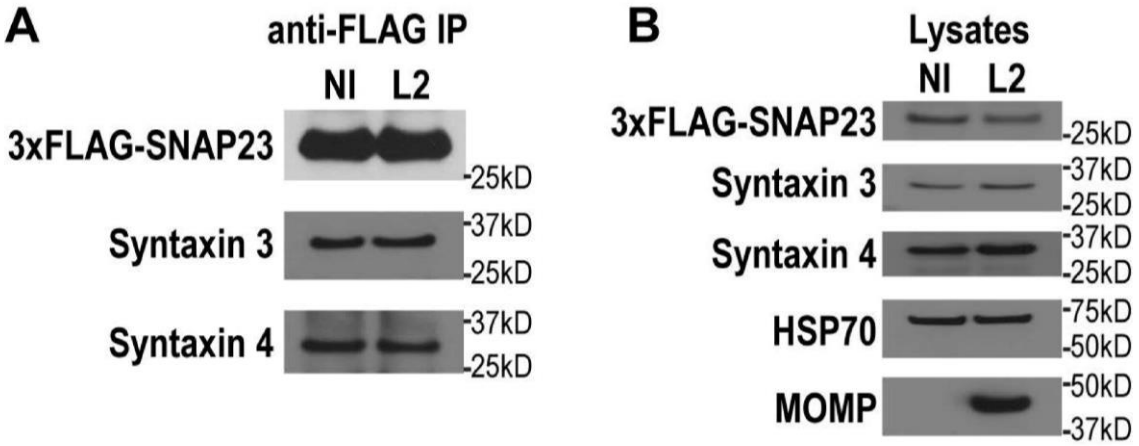

FIGURE S2: *Chlamydia* infection does not impact the interaction between SNAP-23 and its heavy chains Syntaxin 3 and Syntaxin 4. HeLa cells were infected and transfected as in Figure 1. At 24 h pi the cells were treated with N-ethylmaleimide at 4°C to prevent SNARE complex dissociation during lysis. The samples were then subjected to immunoprecipitation (IP) using anti-FLAG antibody. **(A)** The eluates were analyzed by Western blot to probe for interactions between 3xFLAG-SNAP23 and endogenous Syntaxin 3 and Syntaxin 4. **(B)** Cells lysates were analyzed as a control. Anti-HSP70 antibody was used as a HeLa cell loading control. Anti-*C.trachomatis* MOMP was used as an infection control. NI = not-infected; L2 = *C. trachomatis*-infected.

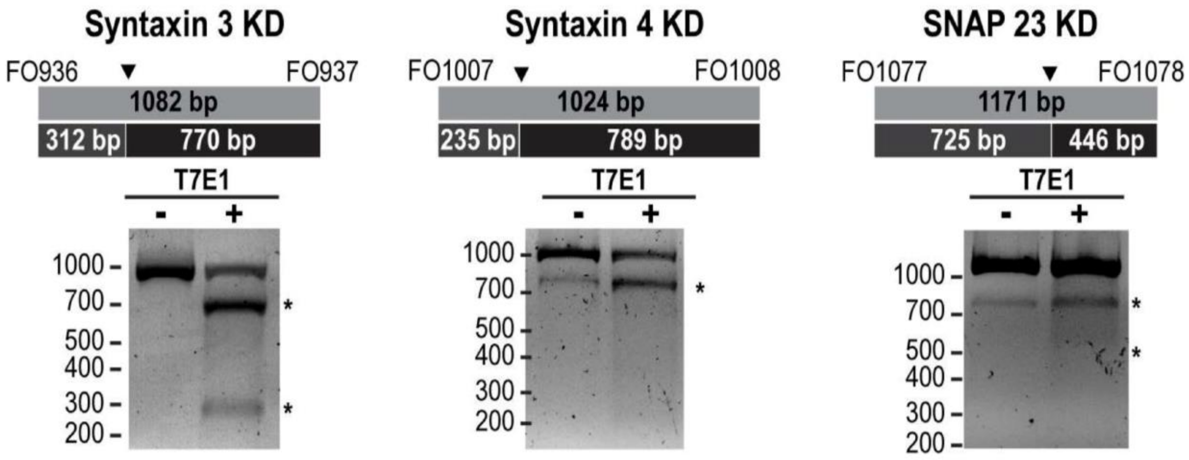

FIGURE S3: Confirmation of Cas9 activity in SNARE knock-down cell lines. HeLa cells were transfected with the different guide-RNAs listed on Table 1 and selected based on their GFP expression. DNA was then extracted for digestion with T7E1 endonuclease and the reactions were analyzed by gel electrophoresis. The predicted sizes of the fragments after T7E1 digestions are shown in the diagrams above the gels. Arrow denotes where T7E1 was predicted to cut if Cas9 cut the genomic DNA as expected. For each cell line, specific primers listed on Table S1 were used for amplification and digestion by T7E1. The fragments indicated by asterisks correspond to the predicted DNA fragments if Cas9 cut at the intended site. (-) and (+) represent the addition of T7E1 endonuclease.

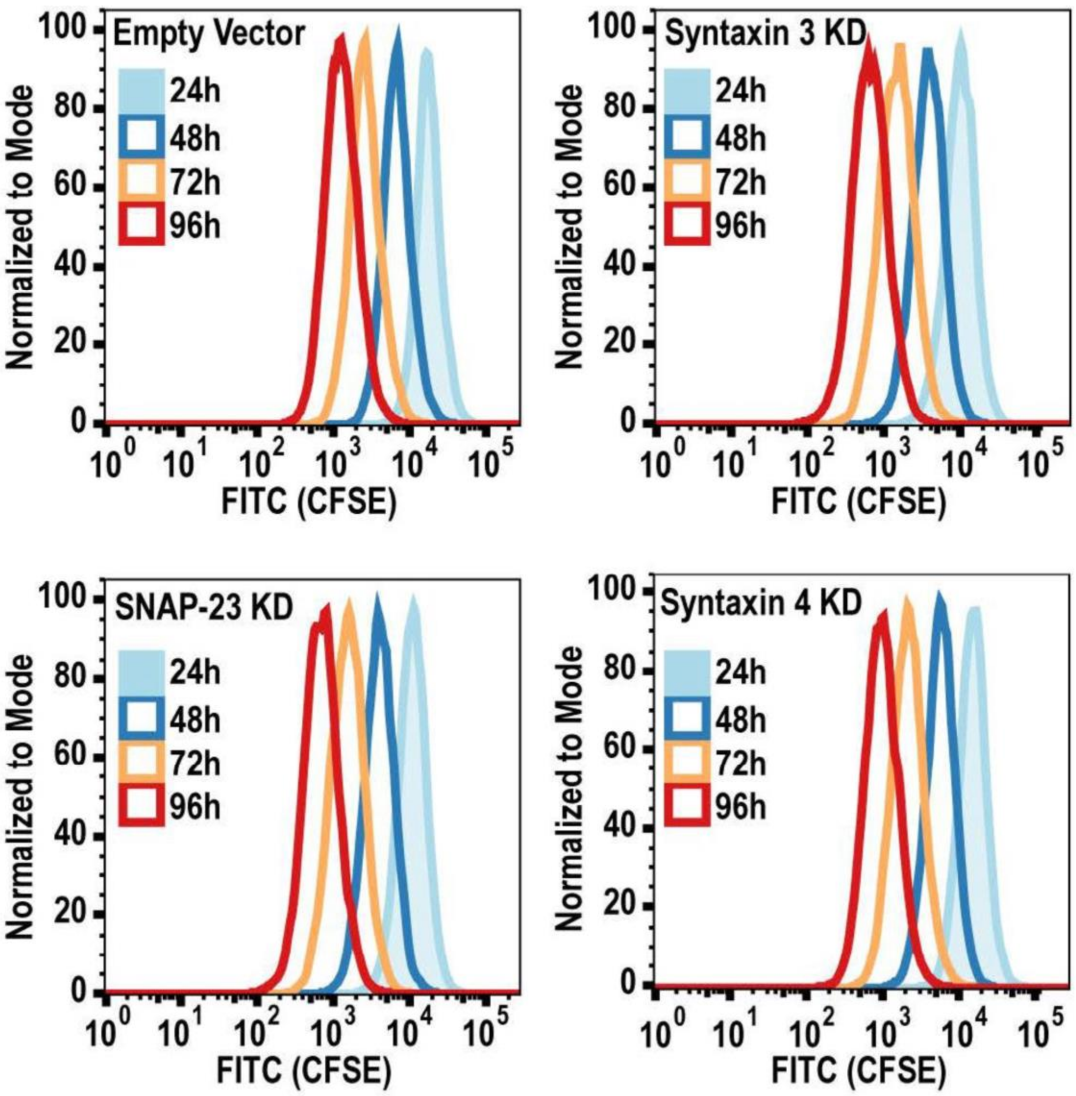

Relative CFSE intensity (% of 24h)				
Time	Empty vector	SNAP-23 KO	Syntaxin 3 KO	Syntaxin 4 KO
24h	100%	100%	100%	100%
48h	39.01%	37.25%	38.59%	38.2%
72h	15.03%	14.32%	15%	14.33%
96h	7.34%	6.52%	6.71%	6.56%

FIGURE S4: Depletion of SNAP-23, Syntaxin 3, and Syntaxin 4 does not affect cell division. Cells were stained with CFSE and incubated at 37°C for 24 h, 48 h, 72 h and 96 h. At each timepoint the CFSE intensity of each sample was measured by flow cytometry. KD = knock down. Table displays the relative CFSE intensity of each cell line normalized to T = 24 h.

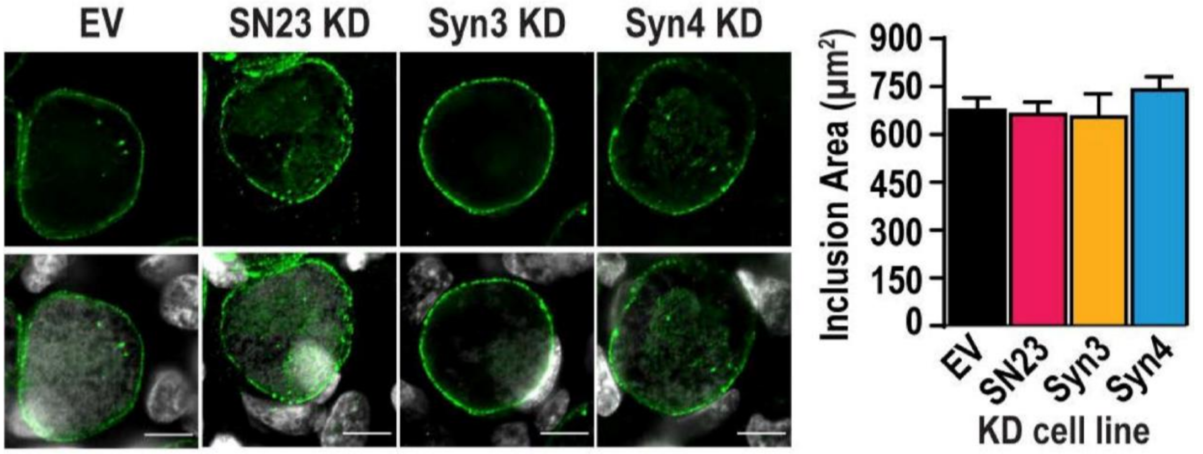

FIGURE S5: SNAP-23, Syntaxin 3, and Syntaxin 4 are not required for inclusion development during infection. The KD cell lines were infected with *C. trachomatis* L2 at a MOI of 0.5 for 46 h, then fixed and stained with anti-IncA antibody (green). Hoechst was used to label DNA (gray). Scale bar = 10 µm. Graph displays the average inclusion area from three independent experiments ± the standard deviation. EV = empty vector control; SN23 = SNAP-23, Syn3 = Syntaxin 3, Syn4 = Syntaxin 4, KD = knock down.

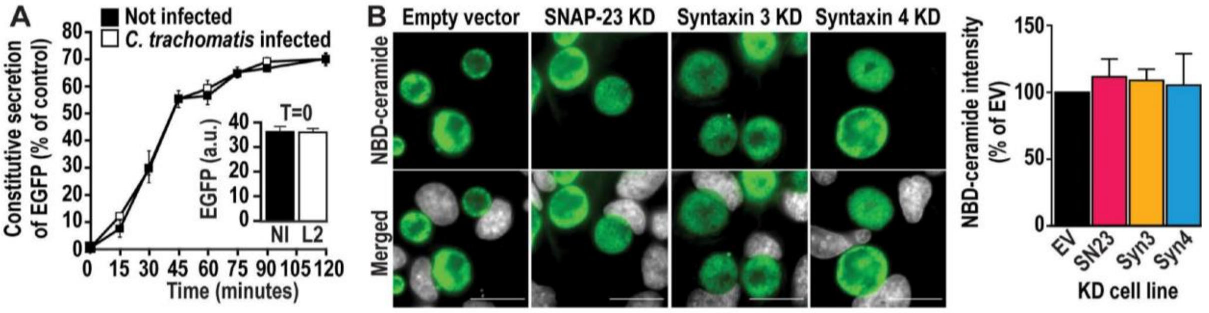

FIGURE S6: SNAP-23 and Syntaxin 4 play a non-canonical role during *Chlamydia* infection. (A) Constitutive secretion is unaffected by *C. trachomatis* infection. HeLa C1 cells were infected with *C. trachomatis* L2 at a MOI of 2 for 22 h. DMEM was added to the non-infected wells as a control. Constitutive secretion of EGFP-hGH was measured for 2 h following the addition of 0.7 µM D/D solubilizer. The EGFP intensity at the given time points was measured and subtracted from the unstimulated wells to obtain the percent secretion. The inset depicts the 100% EGFP signal from the uninduced cells (ethanol). NI = non-infected cells. L2 = WT *C. trachomatis* L2-infected cells. The graph denotes the average of four independent experiments ± the standard deviation. **(B)** SNAP-23, Syntaxin 3, and Syntaxin 4 are not required for ceramide acquisition. KD and empty vector control cells were infected with *C. trachomatis* at a MOI of 1 for 20 h prior to being labeled with NBD-C_6-ceramide (green). Following 6 h of back-exchange, samples were analyzed by immunofluorescence microscopy. Hoechst was used to label DNA (gray). EV = empty vector control, SN23 = SNAP-23, Syn3 = Syntaxin 3, Syn4 = Syntaxin 4, KD = knock down. Scale bar = 20 µm. *Graph*- Graph represents the average NBD fluorescence from the mCherry-positive population from three independent experiments ± the standard deviation. Values were normalized to the EV control.

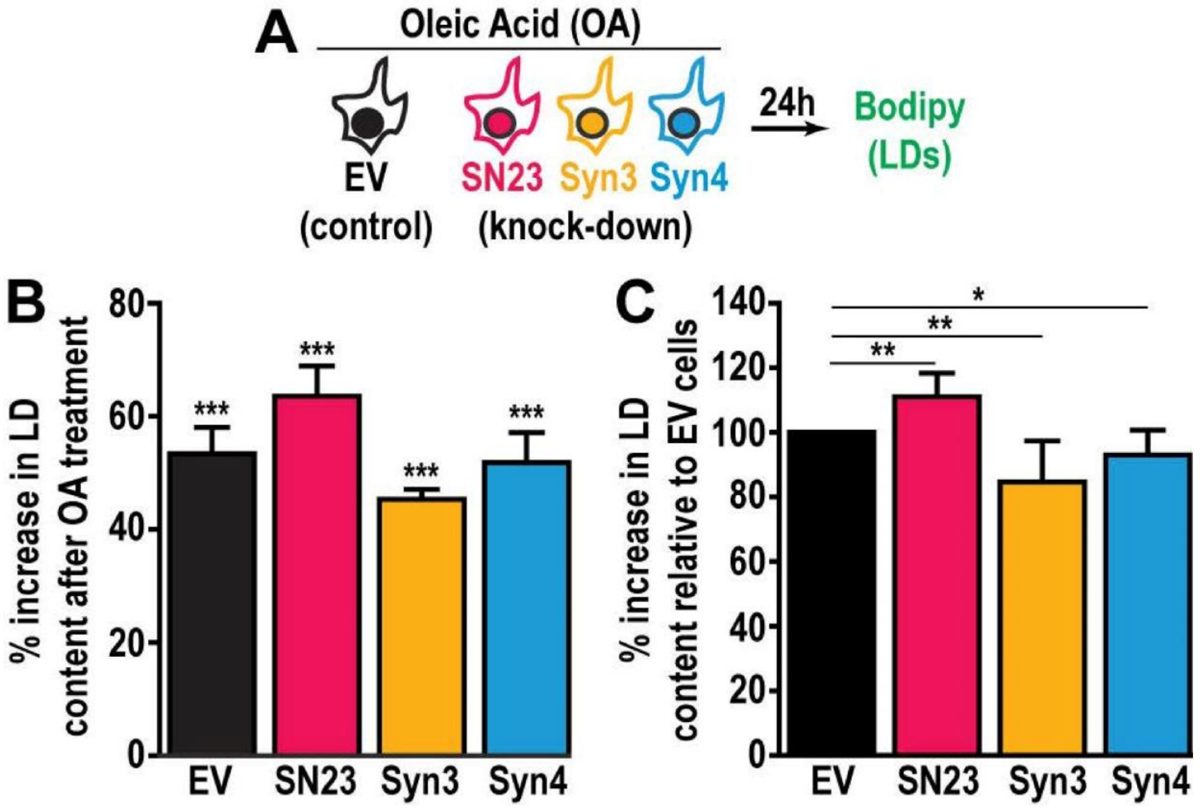

FIGURE S7: OA treatment comparably increases LD content in all of the cell lines. (A) Experimental design. Cells were treated with 200 µM of OA for 24 h. The cells were then stained with BODIPY and fixed. The BODIPY intensity was measured by flow cytometry. **(B)** Graphs represent the average increase in BODIPY staining *relative to non-treated cells* from at least three independent experiments ± the standard deviation. **(C)** Graphs represent the average increase in BODIPY staining *relative to empty vector control cells* from at least three independent experiments ± the standard deviation. The percent increase in BODIPY staining following OA treatment for the empty vector control cell line was arbitrarily set at 100% and represents 155.37% ± 14.40%. Values for the KD cells were then normalized to the EV control. Asterisk (*) denotes a *p value* < 0.05, (**) denotes a *p value* < 0.01, and (***) denotes a *p value* < 0.001. EV = empty vector control, SN23 = SNAP-23, Syn3 = Syntaxin 3, Syn4 = Syntaxin 4, and LD = lipid droplet.

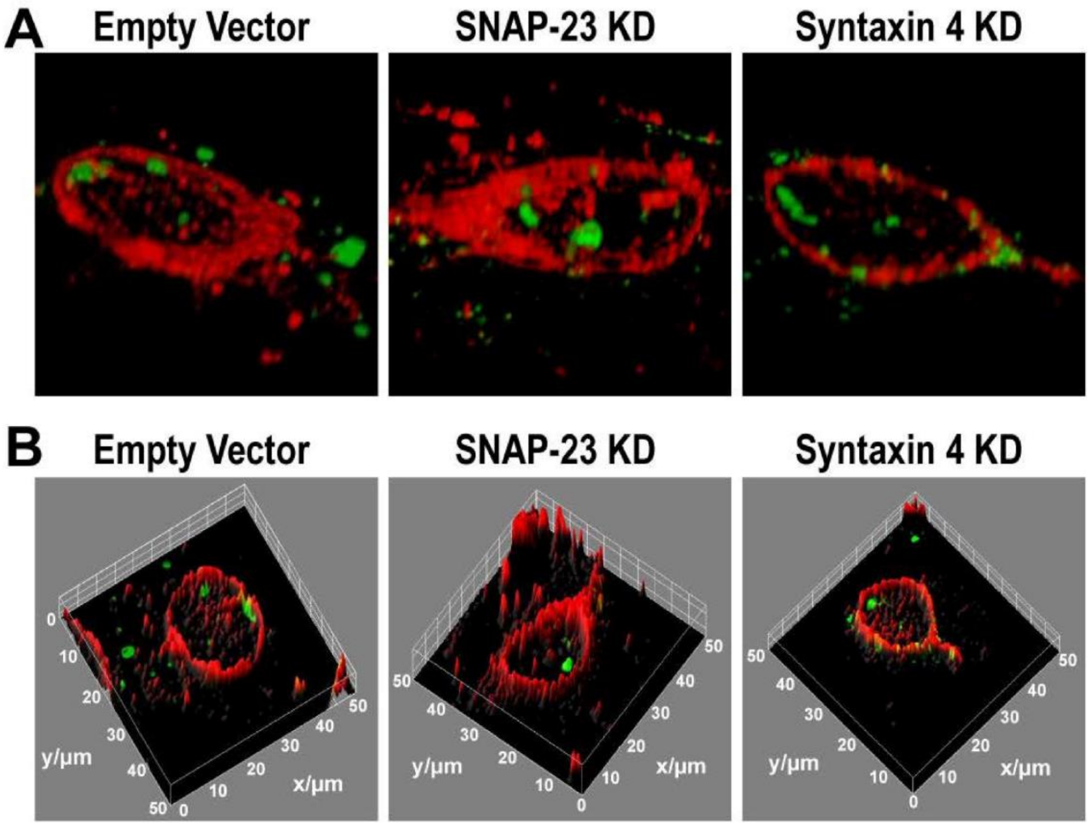

FIGURE S8: LDs are translocated into the inclusion lumen in the absence of SNAP-23 and Syntaxin 4. The cell lines were infected with *C. trachomatis* L2 at a MOI of 0.5 for 30 h. The cells were then fixed and stained with anti-IncA antibody (red) and BODIPY 493/503 (green). **(A)** Three-dimensional reconstruction of IncA (red) and BODIPY (green) channels from the inclusions in the confocal z-stack shown in Figure 3D. **(B)** Three-dimensional surface plot of IncA (red) and BODIPY (green) intensities from the confocal z-stack shown in Figure 3D. KD = knock down.

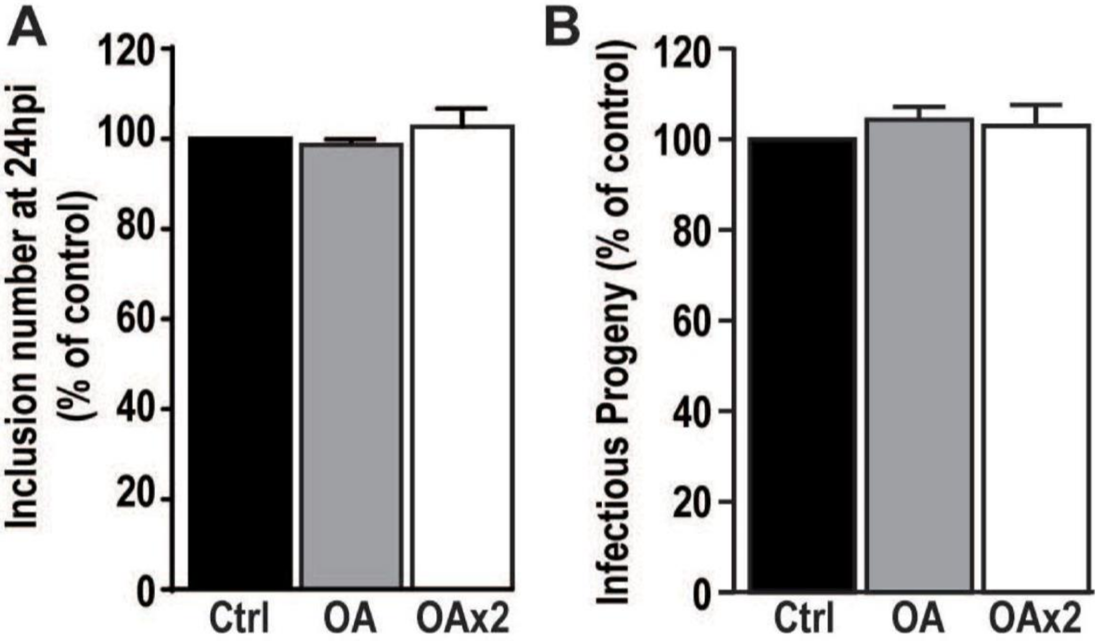

FIGURE S9: Increasing the LD content in HeLa cells using OA prior to infection does not inhibit the generation of infectious progeny. HeLa cells were treated with 200 µM OA at infection (OA) or treated with 200 µM 24 h prior to infection as well as at infection (OAx2) or treated with vehicle (Ctrl). All of the cells were infected with *C. trachomatis* at a MOI of 0.5. **(A)** At 24 hpi, cells were fixed and stained with anti-MOMP antibody. The number of inclusions per well were calculated to determine internalization and early inclusion development, assuming that one inclusion corresponded to an infection started by a single EB. Graph denotes the average of at least three independent experiments ± the standard deviation. For the purpose of comparison, the values obtained for the control were defined arbitrarily as 100 % and represent $3.5 \times 10^5 \pm 1.2 \times 10^4$ inclusion forming units (IFU)/ml. Values for the OA-treated cells were then normalized to the control. **(B)** At 46 h pi, cells were lysed and serially diluted on a fresh monolayer of EV control cells. Twenty-four hours later, the cells were fixed and stained with anti-MOMP antibody. Only dilutions reflecting a MOI < 1 were counted. Graph denotes the average fold change in IFU at T = 46 h compared to T = 0 h of at least three independent experiments ± the standard deviation. The fold change represents how many more IFU were present at T = 46 h compared to T = 0 h. For the purpose of comparison, the values obtained for the control were defined arbitrarily as 100 % and represent a fold change of 1,972.57 ± 163.22. Values for the OA-treated cells were then normalized to the control.

Syntaxin 3 (97.22% identical)

```
  rat MKDRLEQLKA KQLTQDDDTD EVEIAIDNTA FMDEFFSEIE ETRLNIDKIS
human MKDRLEQLKA KQLTQDDDTD AVEIAIDNTA FMDEFFSEIE ETRLNIDKIS

  rat EHVEEAKKLY SIILSAPIPE PKTKDDLEQL TTEIKKRANN VRNKLKSMEK
human EHVEEAKKLY SIILSAPIPE PKTKDDLEQL TTEIKKRANN VRNKLKSMEK

  rat HIEEDEVRSS ADLRIRKSQH SVLSRKFVEV MTKYNEAQVD FRERSKGRIQ
human HIEEDEVRSS ADLRIRKSQH SVLSRKFVEV MTKYNEAQVD FRERSKGRIQ

  rat RQLEITGKKT TDEELEEMLE SGNPAIFTSG IIDSQISKQA LSEIEGRHKD
human RHVEITGKKT TDEELEEMLE SGNPAIFTSG IIDSQISKQA LSEIEGRHKD

  rat IVRLESSIKE LHDMFMDIAM LVENQGEMLD NIELNVMHTV DHVEKARDET
human IVRLESSIKE LHDMFMDIAM LVENQGEMLD NIELNVMHTV DHVEKARDES

  rat KRAMKYQGQA RKKLIIIIVI VVVLLGILAL IIGLSVGLK
human KKAVKYQSQA RKKLIIIIVL VVVLLGILAL IIGLSVGLN
```

Syntaxin 4 (89.23% identical)

```
  rat MRDRTHELRQ GDNISDDEDE VRVALVVHSG AARLSSPDDE FFQKVQTIRQ
human MRDRTHELRQ GDDSSDEEDK ERVALVVHPG TARLGSPDEE FFHKVRTIRQ

  rat TMAKLESKVR ELEKQQVTIL ATPLPEESMK QGLQNLREEI KQLGREVRAQ
human TIVKLGNKVQ ELEKQQVTIL ATPLPEESMK QELQNLRDEI KQLGREIRLQ

  rat LKAIEPQKEE ADENYNSVNT RMKKTQHGVL SQQFVELINK CNSMQSEYRE
human LKAIEPQKEE ADENYNSVNT RMRKTQHGVL SQQFVELINK CNSMQSEYRE

  rat KNVERIRRQL KITNAGMVSD EELEQMLDSG QSEVFVSNIL KDTQVTRQAL
human KNVERIRRQL KITNAGMVSD EELEQMLDSG QSEVFVSNIL KDTQVTRQAL

  rat NEISARHSEI QQLERTIREL HEIFTFLATE VEMQGEMINR IEKNILSSAD
human NEISARHSEI QQLERSIREL HDIFTFLATE VEMQGEMINR IEKNILSSAD

  rat YVERGQEHVK IALENQKKAR KKKVMIAICV SVTVLILAVI IGITITVG
human YVERGQEHVK TALENQKKAR KKKVLIAICV SITVVLLAVI IGVTV-VG
```

FIGURE S10: Protein alignment for rat and human Syntaxin 3 and Syntaxin 4. Amino acid differences are indicated in red. There is 97.22% and 89.23% identity between species for Syntaxin 3 and Syntaxin 4, respectively.

TABLE S1. List of Primers.

NAME	SEQUENCE (5' – 3')	CONSTRUCT
FO822	CACCGGATCTCAGAACATGTAGAGG	Syntaxin 3 knock down gRNA
FO823	AAACCCTCTACATGTTCTGAGATCC	
FO849	AAAAAAAGCACCGACTCGGTGCC	Sequencing primer for the Cas9-GFP vector
FO936	TGGCCTGAATCCTTTCATCCAA	Syntaxin 3 knock down PCR for T7E1 assay
FO937	AGGAACCAAAAATATAGCCCTAAAC	
FO944	CACCGAGAACGTGGAGCGGATTCGG	Syntaxin 4 knock down gRNA
FO945	AAACCCGAATCCGCTCCACGTTCTC	
FO1007	CCTGTCAAGGTCATCACAAGGTG	Syntaxin 4 knock down PCR for T7E1 assay
FO1008	TCTCGGGGTACAGCTAAGGA	
FO1029	CACCGCCTAGTCTCTGGAAAGTACG	SNAP-23 knock down gRNA
FO1030	AAACCGTACTTTCCAGAGACTAGGC	
FO1077	TTCCCATGAACTTCACCATCCAAAT	SNAP-23 knock down PCR for T7E1 assay
FO1078	CCTGCATCCTGAGACTAAGACA	
FO1296	CGGAATTCATGGATAATCTGTCATCAGAAGAAAT C	3xFLAG-SNAP-23
FO1294	CCGCTCGAGTTAGCTGTCAATGAGTTTCTTTGC	
FO1327	CGGAATTCATGAAGGACCGACTGGAGCAGCTGAAGG	3xFLAG-Syntaxin 3
FO1328	CCGCTCGAGTTATTTCAGCCCAACGGACAATCC	
FO1329	CGGAATTCATGCGCGACAGGACCCATGAGTTGAGGC	3xFLAG-Syntaxin 4
FO1330	CCGCTCGAGTTATCCAACGGTTATGGTGATGCC	
